# Unmasking a Silent Killer and Understanding Sudden Cardiac Death in Brugada Syndrome: A Traditional Review

**DOI:** 10.7759/cureus.41076

**Published:** 2023-06-28

**Authors:** Aadya Moturu, Hamsa Priya Bhuchakra, Yashvant P Bodar, Siddharth Kamal Gandhi, Priyansh Patel, Sai Dheeraj Gutlapalli, Vaithilingam Arulthasan, Philip Otterbeck

**Affiliations:** 1 Department of Internal Medicine, Sri Ramaswamy Memorial Medical College Hospital and Research Centre, Chennai, IND; 2 Department of Internal Medicine, Apollo Institute of Medical Sciences and Research, Hyderabad, IND; 3 Department of Internal Medicine, Orenburg State Medical University, Orenburg, RUS; 4 Department of Internal Medicine, M.P. Shah Government Medical College, Jamnagar, IND; 5 Department of Internal Medicine, California Institute of Behavioral Neurosciences & Psychology, Fairfield, USA; 6 Department of Internal Medicine, Medical College Baroda, Vadodara, IND; 7 Department of Internal Medicine, Richmond University Medical Center, New York City, USA

**Keywords:** life-threatening arrhythmia, implantable cardioverter-defibrillator (icd), cardiology research, preventative cardiology, sudden cardiac death (scd)

## Abstract

Brugada syndrome (BrS) is an intricate and heterogeneous genetic disorder that engenders a formidable risk of life-threatening ventricular arrhythmias (VAs). While initially regarded as an electrophysiological aberration, emergent studies have illuminated the presence of underlying structural anomalies in select BrS cases. Although mutations in the SCN5A gene encoding the α-subunit of the cardiac sodium channel were originally identified as a primary causative factor; they account for only a fraction of the syndrome's multifaceted complexity pointing at genetic heterogeneity as a contributing factor. Remarkably, BrS has been linked to a higher incidence of fatal arrhythmic incidents and sudden cardiac death (SCD) with about 4% of SCD cases thought to be caused by BrS. Patients who spontaneously exhibit type one Brugada ECGs are more likely to experience cardiac events, emphasizing the importance of early risk stratification. To aid in risk stratification, the Shanghai score; a multifactorial risk stratification scoring system that incorporates ECG, clinical history, family history, and genetic test results; is utilized to identify those most susceptible to SCD. Beyond single ECGs, evaluation of arrhythmic findings from 24-hour Holter monitoring, ECG variables, electrophysiologic study (EPS) status in the temporal domain, and EPS data collected over time are all critical factors in risk classification. Among management options avoidance of triggers, early risk stratification, and implantation of an Implantable Cardioverter-Defibrillator (ICD) are recommended for asymptomatic patients. For symptomatic patients, pharmacotherapy and ICD implantation are available, with the latter being a highly effective choice for treating and preventing lethal arrhythmias in BrS.

## Introduction and background

Brugada syndrome (BrS) is a silent killer; lurking in the heart's electrical system, waiting to strike without warning. It is a genetic disorder inherited in an autosomal dominant pattern that increases the risk of sudden cardiac death (SCD) and ventricular arrhythmias (VAs) [1]. It is recognized through electrocardiographic (ECG) observations of either apparent or actual right bundle branch block and elevation of the ST-segment in the right precordial leads. These findings are observed in the absence of prolonged QT intervals and structural heart disease [2]. Since 1992, BrS has been recognized as a clinical entity, most found in the South‐East Asia population at 3.7 per 1,000 and predominantly affects younger males [3]. Patients with this condition can exhibit various symptoms, including palpitations, episodes of fainting, or even surviving SCD. Alternatively, some individuals may not experience any noticeable symptoms and remain asymptomatic. The syndrome's three characteristic ECG patterns can occur spontaneously or after exposure to sodium channel-blocking agents [3,4]. SCN5A is a gene located at 3p21 that encodes the pore-forming subunit of the cardiac sodium channel. Around 20% of cases of the disease have been associated with the presence of loss-of-function mutations in this gene [5]. Initially, BrS was speculated to be caused by abnormal repolarization in the right ventricular outflow tract (RVOT) due to inconsistent loss of cardiomyocyte action potential dome in the epicardium. However, further studies using electrophysiological, imaging, and histopathological approaches have detected subtle structural abnormalities in BrS patients including myocardial fibrosis [6]. In patients with BrS, risk stratification is crucial due to two primary reasons: firstly, most patients with the characteristic Brugada ECG pattern are young and asymptomatic, and secondly, an implantable cardioverter-defibrillator (ICD) is the only proven treatment for BrS but ICD is not a benign procedure and can cause significant morbidity [7]. Although most patients undergoing anesthesia, surgery, or invasive procedures for BrS have uncomplicated courses, there is still a risk of exacerbating ST elevation and VA due to various factors such as perioperative medications, surgical trauma, electrolyte imbalances, fever, autonomic nervous system tone, and other perturbations [8]. The most worrisome manifestation of BrS is SCD, which accounts for 4% of all SCD cases; 20% of SCD cases are in patients without structural heart disease [8]. VA is most frequently observed during sleep. Individuals with a history of SCD have an 11-fold higher risk of arrhythmic events compared to asymptomatic individuals [8]. The diagnosis of BrS is crucial for physicians because SCD can be one of the first symptoms to appear in the emergency room and is nearly entirely determined by ECG abnormalities [2]. The most challenging part for the current clinicians is to link the association of SCD to BrS alongside identifying risk factors and ECG patterns. This literature review aims to provide an overview of the current knowledge on the epidemiology, pathophysiology, clinical presentation, diagnosis, and management of SCD in BrS.

Methodology

In conjunction with all authors, PubMed Central, MEDLINE, and PubMed databases were searched. We searched using regular keywords and medical subject headings (MeSH). The following search strategy was selected based on the MeSH vocabulary: (“Death, Sudden, Cardiac” [Majr]) AND (“Brugada Syndrome/complications” [Majr] OR “Brugada Syndrome/mortality” [Majr]). No time limits were set. A total of 1,124 articles were found. Publications that were duplicates or unrelated to humans were immediately excluded. Articles were screened for title and abstract to select studies for free full-text reviews. Each article was screened, and disagreements were discussed among all the authors until consensus was achieved. After a quality appraisal of the studies, a total of 17 studies were finalized to be included in the review. Of those 17 studies, seven were observational studies, five were review studies, three were case reports and two were meta-analyses.

## Review

Pathophysiology

**Figure 1:**
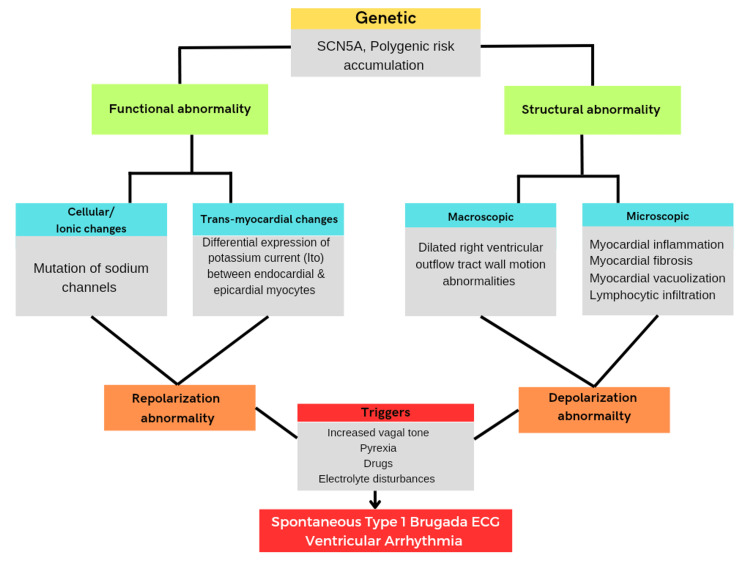
Pathophysiology of Brugada syndrome. ECG: Electrocardiogram. Image credits: Aadya Moturu, Hamsa Priya Bhuchakra and Yashvant P. Bodar.

BrS, initially considered an electrophysiological disorder, produced by dysfunction of a cardiac ion channel in a structurally normal heart has been challenged over time as more studies showed the presence of structural abnormalities in some BrS patients [3]. BrS is a genetic disorder inherited via autosomal dominant transmission. Jellins et al. said that the syndrome was initially linked to mutations in the SCN5A gene responsible for producing the α-subunit of the cardiac sodium channel. However, these mutations are detected in only approximately 18% to 30% of Brugada cases [3]. Moreover, it was noted that most of these mutations are found in familial cases rather than sporadic cases, indicating a diverse range of genetic factors contributing to the syndrome. Other genes such as GPD1-L and KCNE3 have also been linked to the syndrome [3]. The mutations in these genes cause reductions in inward sodium current or gain-of-function in potassium current leading to the overall electrophysiological effect of reduced transmembrane sodium current and increased susceptibility to VA [3]. Bruikman et al. tested the hypothesis that BrS is prevalent among families with premature atherosclerosis (PreAS) in which SCD occurred and concluded that BrS has comparable occurrence rates in families with both SCD and PreAS, as in those with only SCD. SCD in families with both SCD and PreAS appears in more family members and at a later age. These findings suggest that the SCD observed in families with PreAS may be linked to an inherent genetic tendency toward arrhythmias but with a different genetic basis [9]. The pathophysiological mechanism of BrS remains elusive and further two hypotheses have been proposed by Jellins et al., the depolarization theory and the repolarization theory. The repolarization hypothesis suggests that the ST-segment elevation and susceptibility to VAs in BrS are caused by an increased action potential notch in the outer layer of the right ventricle, resulting from a dispersion of repolarization across the heart wall. On the other hand, the depolarization hypothesis proposes that a delay in the conduction of electrical signals in the RVOT leads to delayed development of the action potential. This delay contributes to the characteristic ECG pattern of BrS and creates a substrate for the formation of arrhythmias through a re-entry circuit [3]. A case report conducted by Yorgun et al. on BrS had some fascinating findings. The syndrome was identified after an individual suffered from SCD caused by low potassium levels in the blood due to the consumption of an herbal product, licorice. The significance of examining a patient's use of herbal medicine and its potential to cause electrolyte imbalances and trigger dangerous heart arrhythmias in BrS is emphasized [2]. Contradictory to what Juang et al. said, BrS occurs in the absence of structural heart disease [10]; Jellins et al. stated that BrS has been linked to structural abnormalities in the heart including fibrosis, myocarditis, and enlargement of the right ventricle. The progression of these abnormalities might depend on time, and recent research indicates that the embryological development of the right ventricle could play a role [3]. In a study conducted by Miles et al., it was discovered that individuals with BrS have higher levels of collagen throughout the myocardium of both the right and left ventricles, particularly in the thin-walled epicardium of the RVOT. This increase in collagen may contribute to the formation of abnormal tissue and disruptions in conduction, leading to arrhythmias in BrS. The study did not find significant variations in the proportion of myocardial fat between BrS patients and control subjects. However, it did observe specific differences related to age and sex, with older individuals and females having a higher proportion of myocardial fat [8]. In a study conducted by Nademanee et al., the authors observed a reduction in the Cx43 signal in the myocardium of patients with BrS compared to the control myocardium. This suggests that alterations in Cx43 expression at the intercalated disc could lead to an electrical disconnection between cardiomyocytes, which may play a significant role in the development of BrS [6]. The exact cause of these structural alterations is still unknown and a unifying theory for the syndrome has yet to be established [3]. BrS displays a wide range of clinical manifestations, and syncopal episodes or cardiac arrest due to arrhythmic complications like polymorphic ventricular tachycardia (VT) or ventricular fibrillation (VF) occur in approximately 20% to 40% of diagnosed patients. Supraventricular tachycardias (SVT) have been observed in up to one-fifth of individuals with BrS [3]. In a study by Morita et al., a significant proportion (39%) of patients with BrS experienced spontaneous atrial fibrillation (AF), and they were found to be more susceptible to AF following electrical stimulation [11]. Conversely, Takigawa et al. suggested that symptoms in BrS tend to exhibit prominent peaks during specific times, with a peak during the late night to early morning hours and another peak during the spring to the early summer season [12]. It should be noted that asymptomatic patients may remain undiagnosed which may result in an overestimation of the frequency of symptoms [3]. The syndrome is significantly more common in Asia compared to Western populations, with a five-fold difference in prevalence. It is mostly found in Southeast Asia, followed by North Africa, the Middle East, East Asia, South Asia, North America, Europe, and Hawaii, with the highest prevalence observed in those regions [13]. According to Jellins et al., BrS is more prevalent in men than in women, with an estimated ratio of eight to 10 men for every affected woman. This higher occurrence in men is attributed to the significantly higher density of Ito currents in males compared to females. This disparity in Ito currents increases the vulnerability of men to develop ventricular arrhythmias [3]. Shimizu et al. and Bai et al. suggest that hormones may play a role in the gender differences observed in BrS with clinical and experimental studies indicating a possible hormonal influence on ion currents [14,15]. As opposed to this, Probst et al. did not find a higher prevalence of syndrome in males compared to females in the pediatric population [16]. The absence of gender differences in BrS may be attributed to lower levels of testosterone found in children of both sexes [3]. According to a study by Rattanawong et al., individuals with a family history of SCD in relatives younger than 40 years old are at approximately twice the risk of major adverse events in the presence of BrS [17]. Jellins et al. showed that BrS is characterized by three distinct ECG subtypes that can be identified in the right precordial leads (V1-V3). The type one ECG pattern, which is diagnostic of BrS, is characterized by a coved-shaped ST-segment elevation greater than 2 mm, followed by an inverted T-wave in one or more of the right precordial leads. The type two pattern exhibits an ST-segment resembling a saddleback, while the type three pattern displays either a coved or saddleback ST-segment elevation ranging from 1 mm to 2 mm. Although type two and type three ECG findings can occur spontaneously, they are not considered diagnostic. However, if these patterns convert to a type one ECG pattern after provocation using a sodium channel blocking agent, and at least one of the characteristic clinical findings is present, a diagnosis of BrS can be considered. Serial ECG recordings are recommended due to expected variability [3]. The pathophysiology of BrS is explained in Figure 1.

Complications and risk stratification

Miles et al. noted that BrS has been associated with an increased risk of serious arrhythmic events and SCD as well as additional consequences such as cardiomyopathy and lethal syncope. The most concerning symptom of BrS is SCD [8]. According to a study conducted by Zhu et al., BrS, also known as unexplainable sudden death syndrome, is the primary cause of sudden death in young people and affects as many as four to 10 individuals per 10,000 in the general population. The findings from this epidemiological study indicate that BrS is responsible for SCD in nearly 50% of young individuals in South Asia who have nonstructural heart disease [18]. The syndrome is likely to be the cause of 4% of all SCD cases and 20% of SCD patients who do not have structural heart disease. As noted by Sorajja et al., individuals with a history of SCD are at an 11-fold higher risk of experiencing arrhythmic events compared to asymptomatic individuals [1]. A meta-analysis conducted by Rattanawong et al. revealed the presence of SCD history in younger family members, indicating a higher incidence of major arrhythmic events in younger patients with BrS when stratified by age groups. However, a pooled analysis did not find a significant association between the presence of a family history of SCD without specific age criteria in BrS and major arrhythmic events [17]. Lethal cardiac tachyarrhythmias like VF, which have both acquired and hereditary causes as well as a combination of both, are the most common cause of SCD [17]. According to a meta-analysis conducted by Wu et al., patients with a history of SCD or syncope were found to have a 4.97-fold increased risk of experiencing cardiac events. Additionally, individuals who displayed inducible VA during an electrophysiological study (EPS) had a 3.56-fold higher risk of cardiac events. Additionally, patients who had spontaneous type one Brugada ECGs were 2.78 times more likely to experience cardiac events [19]. According to research by Lee et al., spontaneous VT/VF in BrS was predicted by P wave duration, the existence of other arrhythmias such as AF, mean QRS duration, QTc intervals as well as other factors [13]. By using Kaplan-Meier analyses, Tokioka et al. demonstrated that the combination of f-QRS (abnormal depolarization) and ER (abnormal repolarization) is useful for predicting VF events in patients with BrS, demonstrating the significance of the combination of depolarization and repolarization in the development of lethal arrhythmia [20]. Concomitant disorders appear to be associated with an increased risk of SCD in BrS, as they share the same key electrophysiological mechanism, i.e., reduced cardiac excitability [1]. Some families with a history of BrS exhibit diverse and discordant phenotypes among their members, posing a diagnostic and risk stratification challenge to physicians. Among these early repolarization syndrome (ERS), progressive cardiac conduction disease (PCCD, Lev-Lenègre syndrome), and sick sinus syndrome (SSS) are a few conditions linked to BrS [21]. Bruikman et al. noted that a subset of BrS patients, approximately 20%, also experience supraventricular arrhythmias including atrial flutter, AF, and preexcitation syndromes like Wolff-Parkinson-White syndrome, as reported in a study [9]. While life-threatening VA are typically associated with BrS, there is increasing recognition of atrial arrhythmias, particularly AF, among patients with BrS [4]. According to a meta-analysis by Kewcharoen et al., these atrial arrhythmias are estimated to be present in 10%-20% of individuals with BrS [4]. A territory-wide retrospective cohort study in Hong Kong by Lee et al. observed that atrial arrhythmias and abnormalities in ventricular repolarization are additional factors that influence the development of ventricular arrhythmogenesis in BrS and ECG biomarkers that reflect these processes and offer additional value for risk assessment [13]. Bruikman et al. discovered BrS in 16% of families with unexplained SCD (with or without PreAS) at age >45 years, as well as a prevalence of 20% of a cardiac conduction disease in families as suggested by positive ajmaline tests and 18% of families, had SCN5A mutation with SCD alone [9]. Bayrak et al. observed the risk stratification of BrS to be aimed at identifying individuals most liable to SCD so that they can receive appropriate management. BrS is clearly more prevalent in males, and it is associated with a threefold higher likelihood of exhibiting a type one electrocardiogram (ECG) pattern and/or experiencing a cardiac event, according to a study [21]. However, in women, identifying conduction disturbances and sinus dysfunction may serve as better indicators of risk compared to the presence of a spontaneous type one ECG pattern and associated symptoms. In children, BrS-related SCD is uncommon. Although the risk can be substantial, especially in kids who have had symptoms in the past and have a spontaneous ECG pattern [21]. Zhu et al. hypothesized that individuals with BrS may present with or without symptoms based on a spontaneous or incited type one ECG pattern. Aborted SCD, syncope, seizures, and nocturnal agonal breathing are among the symptomatic manifestations. Malignant syncope can increase the risk of SCD in people who have spontaneous type one Brugada waves [18]. The risk of SCD is approximately four times higher in persons with a history of syncope than in those with no history of syncope. In contrast, the clinical course of asymptomatic people with spontaneous type one Brugada waves is typically benign [18]. People without symptoms should have their family history reviewed. Finding a spontaneous BrS pattern on an ECG has consistently been linked to a higher risk of SCD, ranging from 0.81% per year in asymptomatic patients to 2.33% per year in symptomatic patients [22]. Shanghai score is a risk stratification scoring method that combines ECG, clinical history, family history, and genetic test results, with a score of 3.5 or higher indicating probable/definite BrS [23]. Nakano et al. noted that VT/VF episodes increased in frequency as the score increased and that no patient with a score of 3.5 or lower experienced VF events. The study's findings supported the Shanghai score's validity and demonstrated the significance of a thorough diagnosis of BrS [23]. The assessment of ECG variables and EPS status in the temporal domain, arrhythmic findings in 24-hour Holter monitoring, and EPS data obtained over time all contribute to risk stratification in addition to single ECGs [13]. According to Lee et al., EPS is the most employed test for assessing the risk of arrhythmias in BrS and other conditions associated with increased arrhythmia susceptibility. EPS is a valuable tool for predicting future episodes of VT/VF and plays a crucial role in the risk stratification of BrS [13].

Prevention and treatment

To prevent BrS-associated SCD, early identifying BrS with meticulous history taking, lab test for potassium-calcium balance, EPS assessment and genetic testing is important. Accurate identification of asymptomatic patients who are at risk of developing lethal arrhythmia in BrS is a challenge, and there are currently limited treatment options available for these individuals. However, there are some strategies that can be employed to identify high-risk individuals, such as genetic testing and ECG screening, although these approaches have some limitations [3]. In addition to identifying high-risk individuals, lifestyle modifications can play a role in preventing lethal arrhythmia in BrS. For example, avoidance of triggers such as fever, dehydration, and certain medications (such as antiarrhythmics (Class IA and IC), anesthetic agents, psychiatric drugs, and substance abuse) can be helpful in reducing the risk of arrhythmias. Furthermore, careful monitoring and management of electrolyte imbalances and other medical conditions that may exacerbate the syndrome can also be beneficial [1]. While drug therapy has not been shown to be effective in preventing lethal arrhythmia in BrS, some medications such as beta-blockers and sodium channel blockers may be used to manage symptoms and reduce the risk of arrhythmia in some patients. However, the use of these medications should be carefully considered on a case-by-case basis, as they can also have potential adverse effects. Ultimately, early risk stratification and evaluation for ICD implantation remain the most effective means of preventing lethal arrhythmia in BrS [1]. Close monitoring and follow-up with healthcare providers are essential for all individuals with BrS, including those who are asymptomatic. The pharmaceutical approach is taken into consideration using quinidine. It is used when ICD implantation is not possible or used during recurrent arrhythmias. Quinidine and isoprenaline are also effective in treating complications such as electrical storms or inappropriate shocks with ICDs [1]. According to Belhassen et al., quinidine alone is useful in the treatment of BrS [24]. Corcia et al., on the other hand, said that despite its effectiveness, quinidine is not an ICD substitute [25]. Additionally, long-term use of quinidine is associated with various adverse effects including diarrhea, thrombocytopenia, hepatitis, and pro-arrhythmic activity leading to torsades de pointes. As a result, drug therapy is not a first-line choice of treatment when ICD implantation is possible [3,25]. ICD has shown to be an effective choice in the treatment and prevention of SCD in BrS [25]. According to Corcia et al., ICDs are effective in treating lethal arrhythmias, although there is a potential risk of device-related complications. Therefore, regular follow-up is essential to adjust the ICD configuration based on age and level of activity to minimize inaccurate shocks and device-related complications such as ventricular electrode fracture, lead dislocation, and pulse generator migration [25]. In contrast, patients with ICDs who are undergoing surgery need to change their ICD configuration pre-operatively and reprogram post-operatively to avoid device-related problems such as VA [1]. However, device-related problems have been lessened over the previous few decades as device technology and programming have improved. As a result, ICD is chosen over pharmacological therapy [25].

Limitations

The study has some limitations, including the lack of sufficient high-level evidence, such as randomized controlled trials or meta-analyses. All identified studies were based on the limited number of clinical trials available. All the studies showed heterogeneity in sample size, measuring the variables. Not all the studies assessed have similar variables and secondary outcomes. Only the papers in English were included in this review; hence, information from papers in languages other than English was not included. We only included studies with human subjects; studies with any animal trials were not included in this study.

## Conclusions

Overall, BrS is a complex and heterogenous genetic disorder that can lead to life-threatening arrhythmias and SCD. Early diagnosis and risk stratification are crucial for identifying those at the highest risk and implementing appropriate management strategies. Currently, ICD implantation is the most effective way to prevent fatal arrhythmias in those at high risk. However, further research is needed to better understand the pathophysiology of BrS and develop standardized management protocols. By staying up-to-date with the latest research and guidelines, physicians can provide optimal care for patients with BrS.

## References

[1] Sorajja D, Ramakrishna H, Poterack AK, Shen WK, Mookadam F (2015). Brugada syndrome and its relevance in the perioperative period. Ann Card Anaesth.

[2] Yorgun H, Aksoy H, Sendur MA, Ateş AH, Kaya EB, Aytemir K, Oto A (2010). Brugada syndrome with aborted sudden cardiac death related to liquorice-induced hypokalemia. Med Princ Pract.

[3] Jellins J, Milanovic M, Taitz DJ, Wan SH, Yam PW (2013). Brugada syndrome. Hong Kong Med J.

[4] Kewcharoen J, Rattanawong P, Kanitsoraphan C (2019). Atrial fibrillation and risk of major arrhythmic events in Brugada syndrome: a meta-analysis. Ann Noninvasive Electrocardiol.

[5] Bezzina CR, Barc J, Mizusawa Y (2013). Common variants at SCN5A-SCN10A and HEY2 are associated with Brugada syndrome, a rare disease with high risk of sudden cardiac death. Nat Genet.

[6] Nademanee K, Raju H, de Noronha SV (2015). Fibrosis, connexin-43, and conduction abnormalities in the Brugada syndrome. J Am Coll Cardiol.

[7] Nademanee K, Veerakul G (2014). Overlapping risks of early repolarization and Brugada syndrome. J Am Coll Cardiol.

[8] Miles C, Asimaki A, Ster IC (2021). Biventricular myocardial fibrosis and sudden death in patients with Brugada syndrome. J Am Coll Cardiol.

[9] Bruikman C, de Ronde MW, Amin A (2020). Sudden cardiac death in families with premature cardiovascular disease. Heart.

[10] Juang JMJ, Phan WL, Chen PC (2011). Brugada-type electrocardiogram in the Taiwanese population-is it a risk factor for sudden death. J Formos Med Assoc.

[11] Morita H, Kusano-Fukushima K, Nagase S (2002). Atrial fibrillation and atrial vulnerability in patients with Brugada syndrome. J Am Coll Cardiol.

[12] Takigawa M, Noda T, Shimizu W (2008). Seasonal and circadian distributions of ventricular fibrillation in patients with Brugada syndrome. Heart Rhythm.

[13] Lee S, Zhou J, Li KH (2021). Territory-wide cohort study of Brugada syndrome in Hong Kong: predictors of long-term outcomes using random survival forests and non-negative matrix factorisation. Open Heart.

[14] Shimizu W, Matsuo K, Kokubo Y (2007). Sex hormone and gender difference--role of testosterone on male predominance in Brugada syndrome. J Cardiovasc Electrophysiol.

[15] Bai CX, Kurokawa J, Tamagawa M, Nakaya H, Furukawa T (2005). Nontranscriptional regulation of cardiac repolarization currents by testosterone. Circulation.

[16] Probst V, Denjoy I, Meregalli PG (2007). Clinical aspects and prognosis of Brugada syndrome in children. Circulation.

[17] Rattanawong P, Kewcharoen J, Kanitsoraphan C (2021). Does the age of sudden cardiac death in family members matter in Brugada syndrome. J Am Heart Assoc.

[18] Zhu YB, Zhang JH, Ji YY (2022). Analysis of a family with brugada syndrome and sudden cardiac death caused by a novel mutation of SCN5A. Cardiol Res Pract.

[19] Wu W, Tian L, Ke J, Sun Y, Wu R, Zhu J, Ke Q (2016). Risk factors for cardiac events in patients with Brugada syndrome: a PRISMA-compliant meta-analysis and systematic review. Medicine (Baltimore).

[20] Tokioka K, Kusano KF, Morita H (2014). Electrocardiographic parameters and fatal arrhythmic events in patients with Brugada syndrome: combination of depolarization and repolarization abnormalities. J Am Coll Cardiol.

[21] Bayrak F, Brugada P (2022). Recent status in Brugada syndrome. Turk Kardiyol Dern Ars.

[22] Gourraud JB, Barc J, Thollet A, Le Marec H, Probst V (2017). Brugada syndrome: diagnosis, risk stratification and management. Arch Cardiovasc Dis.

[23] Nakano Y, Shimizu W (2022). Brugada syndrome as a major cause of sudden cardiac death in Asians. JACC Asia.

[24] Belhassen B, Glick A, Viskin S (2004). Efficacy of quinidine in high-risk patients with Brugada syndrome. Circulation.

[25] Gonzalez Corcia MC, Sieira J, Pappaert G (2018). Implantable cardioverter-defibrillators in children and adolescents with Brugada syndrome. J Am Coll Cardiol.

